# Intravascular optical imaging of high-risk plaques *in vivo* by targeting macrophage mannose receptors

**DOI:** 10.1038/srep22608

**Published:** 2016-03-07

**Authors:** Ji Bak Kim, Kyeongsoon Park, Jiheun Ryu, Jae Joong Lee, Min Woo Lee, Han Saem Cho, Hyeong Soo Nam, Ok Kyu Park, Joon Woo Song, Tae Shik Kim, Dong Joo Oh, DaeGab Gweon, Wang-Yuhl Oh, Hongki Yoo, Jin Won Kim

**Affiliations:** 1 Multimodal Imaging and Theranostic Lab, Cardiovascular Center, Korea University Guro Hospital, Seoul, Republic of Korea; 2 Division of Bio-imaging, Chuncheon Center, Korea Basic Science Institute, Republic of Korea; 3 Department of Mechanical Engineering, KAIST, Daejeon, Republic of Korea; 4 Department of Biomedical Engineering, Hanyang University, Seoul, Republic of Korea

## Abstract

Macrophages mediate atheroma expansion and disruption, and denote high-risk arterial plaques. Therefore, they are substantially gaining importance as a diagnostic imaging target for the detection of rupture-prone plaques. Here, we developed an injectable near-infrared fluorescence (NIRF) probe by chemically conjugating thiolated glycol chitosan with cholesteryl chloroformate, NIRF dye (cyanine 5.5 or 7), and maleimide-polyethylene glycol-mannose as mannose receptor binding ligands to specifically target a subset of macrophages abundant in high-risk plaques. This probe showed high affinity to mannose receptors, low toxicity, and allowed the direct visualization of plaque macrophages in murine carotid atheroma. After the scale-up of the MMR-NIRF probe, the administration of the probe facilitated *in vivo* intravascular imaging of plaque inflammation in coronary-sized vessels of atheromatous rabbits using a custom-built dual-modal optical coherence tomography (OCT)-NIRF catheter-based imaging system. This novel imaging approach represents a potential imaging strategy enabling the identification of high-risk plaques *in vivo* and holds promise for future clinical implications.

Atherosclerosis is considered as a chronic inflammatory disease^1^. While most atherosclerotic plaques remain clinically silent, some lesions become rupture-prone, resulting in acute cardiovascular events. Structural aspects including thin-cap fibroatheroma (TCFA) and the inflammation in the fibrous cap are established major discriminators of high-risk plaques causing acute events^2^,^3^. However, current diagnostic modalities providing morphological information are insufficient to predict the coronary risk^4^. Macrophages, which are pivotal contributors to plaque instability through the release of inflammatory precursors such as proteases, reactive oxygen species, and immune mediators^5^,^6^,^7^,^8^, have emerged as a key imaging target for high-risk coronary atheromata^9^,^10^,^11^,^12^,^13^. One promising approach to visualize plaque macrophages has been optical molecular imaging by excitation of near-infrared fluorescence (NIRF) probe targeting inflammatory molecular pathways^14^. We recently constructed an intravascular dual-modal structural-molecular catheter-based imaging system^15^. The complete integration of the NIRF molecular imaging with the three-dimensional comprehensive optical coherence tomography (OCT)^16^ enabled simultaneous visualization of structural and molecular information of atherosclerotic plaques^15^. While the recently upgraded high-speed OCT-NIRF imaging technique using a NIRF-emitting agent, indocyanine green (ICG), was highly translatable for identification of lipid-rich inflamed atheromata *in vivo*^17^,^18^, the nonspecific binding of ICG to both lipids and macrophages has still limited the precise assessment of the molecular contents within atherosclerotic plaques.

Motivated by the deficiencies in targeting molecular pathway, herein, we newly developed a NIRF-emitting optical imaging probe that specifically targets macrophage mannose receptors (MMRs). Since mannose receptors on macrophages are highly expressed in TCFA^19^, and particularly associated with neovascularization and intraplaque hemorrhage^20^,^21^, they are potentially utilizable as a robust imaging target for high-risk plaques^19^. We first evaluated the MMRs with a customized multichannel laser confocal fluorescence microscope in murine carotid plaque *in vivo*. Then, with the scale-up of the MMR-NIRF probe, we applied it for coronary-sized plaque imaging using our intravascular high-speed dual-modal OCT-NIRF imaging system. Finally, we estimated the safety of the MMR-NIRF probe *in vitro* and *in vivo* as well.

## Results

### Synthesis and *In Vitro* Cellular Uptake Study of the MMR-targeting probe

**Figure 1:**
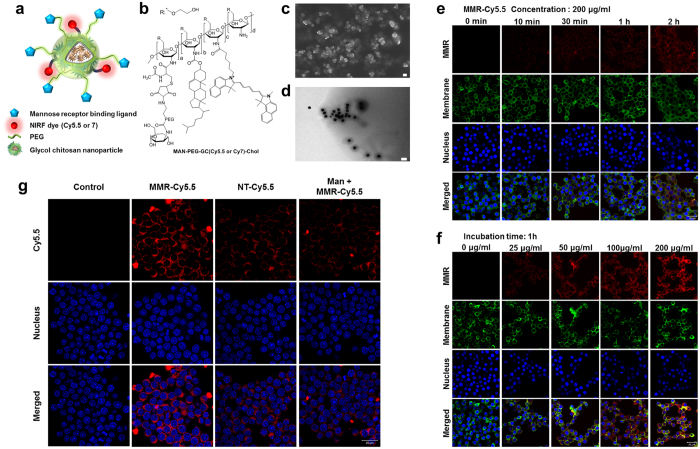
Synthesis of the Macrophage Mannose Receptor (MMR) targeting Nanoprobe and *In Vitro* Uptake. (**a**) Schematic illustration and (**b**) molecular structure of the MMR-targeting nanoprobe. (**c**) SEM (scale bar, 100 nm) and (**d**) TEM (scale bar, 200 nm) images of the MMR-targeting probe. (**e–g**) Confocal fluorescence microscopy images of macrophage cells (RAW264.7) after incubation with MMR-Cy5.5. Fluorescence signals of MMR-Cy5.5 were presented in red. The cell membrane was counterstained with CellMask^TM^ Orange (pseudocolor, green), and the nucleus with DAPI (blue). (**e**) Time-dependent intracellular fluorescence intensity is shown in fluorescence confocal images of macrophage cells incubated with 200 μg/mL of MMR-Cy5.5 for 0 min, 10 min, 30 min, 1 h, and 2 h. Scale bar, 20 μm. (**f**) Concentration-dependent intracellular fluorescence intensity is shown in fluorescence confocal images of macrophage cells incubated with 0, 25, 50, 100, and 200 μg/mL of MMR-Cy5.5 for 1 h. Scale bar, 20 μm. (**g**) Fluorescence intensity in macrophages with no-treatment (1st column), MMR-Cy5.5 (2nd column), NT-Cy5.5 (3rd column), and pre-treatment with free mannosamine (4th column). Fluorescence intensity from macrophage cells could be reduced significantly by incubation with NT-Cy5.5 or pre-treatment with free mannosamine. Scale bar, 20 μm.

The schematic image and chemical structure of the NIRF-emitting probe targeting MMRs are presented in Fig. 1a,b. The MMR-targeting probe was synthesized using thiolated glycol chitosan, mannosamine-polyethylene glycol-maleimide (MAN-PEG-MAL), cholesteryl chloroformate, and cyanine 5.5 (Cy5.5) or cyanine 7 (Cy7) (Supplementary Fig. 1 and Supplementary Fig. 2) and was confirmed with ^1^H nuclear magnetic resonance spectroscopy (^1^H-NMR) (Supplementary Fig. 3). The compound formed self-assembled nanostructures with a diameter range of 50–100 nm, as determined by scanning electron microscopy and transmission electron microscopy (Fig. 1c,d). There were no significant differences between the non-targeting (NT) probes and the MMR-targeting probes with respect to shape, size, and size distribution (Supplementary Fig. 4).

*In vitro* intracellular uptake of Cy5.5-labeled MMR-targeting probes (MMR-Cy5.5) in macrophages was monitored by confocal laser scanning microscopy with respect to treatment time and dose. As the incubation time increased, the fluorescence signal became more intense inside the cells, and evenly distributed in the cytosol (Fig. 1e). Furthermore, the degree of cellular uptake was clearly dose-dependent, which was determined experimentally by increasing the concentration from 25 to 200 μg/mL (Fig. 1f). In order to demonstrate the specific binding affinity of MMR-Cy5.5 to mannose receptors, a blocking study with free mannosamine was performed. The fluorescence intensity of MMR-Cy5.5 was higher than that of the Cy5.5-labeled NT probes (NT-Cy5.5), and the intensity decreased when free mannosamine was pre-treated, indicating that MMR-Cy5.5 can more specifically target mannose receptors compared to the NT-Cy5.5 probe (Fig. 1g).

### Biodistribution, *In Vitro* and *In Vivo* Toxicity Studies of the MMR-Targeting Probe

**Figure 2:**
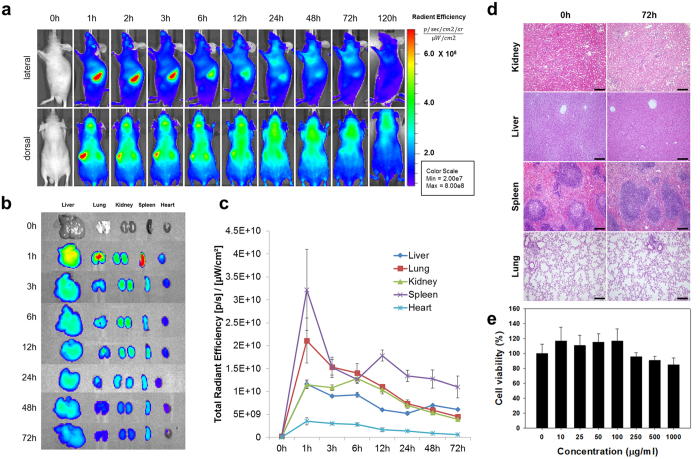
Biodistribution and Toxicity Assay of MMR-Cy5.5. (**a**) *In vivo* fluorescence whole body imaging to evaluate the distribution of MMR-Cy5.5 in C57BL/6 nude mice at different time points after injection. (**b**) *Ex vivo* fluorescence imaging of the harvested liver, lung, kidney, spleen, and heart from MMR-Cy5.5-injected wild type C57BL/6 mice analyzed at scheduled time-points. (**c**) The time dependent changes of fluorescence intensities of isolated organs from *ex vivo* images. (**d**) H&E stained images of the kidney, liver, spleen, and lung from MMR-Cy5.5-injected mice for toxicity analysis. No histopathological changes were observed at 72 h post-injection. Scale bar, 200 μm. (**e**) Cellular toxicity analysis shows that 85–95% of the cells are still viable after incubation with various concentrations of MMR-Cy5.5 up to 1000 μg/mL for 24 h.

Prior to performing the *in vivo* imaging studies, we evaluated the time-dependent excretion and tissue distribution of the MMR-Cy5.5 in C57BL/6 nude mice (n = 5) and wild type C57BL/6 mice (n = 3), respectively (Supplementary Fig. 5a). The time-dependent excretion of MMR-Cy5.5 was clearly visualized by monitoring the fluorescence signal emitted from the entire body (Fig. 2a). At initial time points 1–6 hour post-injection, strong fluorescence signals were observed in the spleen from both lateral and dorsal views. The NIRF signals gradually decreased but remained visible in the dorsal view for up to 2 days, demonstrating a prolonged circulation time of the probe.

Tissue distribution of the MMR-Cy5.5 was evaluated through *ex vivo* NIRF imaging of organs including the liver, lung, kidney, spleen, and heart at scheduled time points (n = 21, 3/each time point) (Supplementary Fig. 5b). Enhanced NIRF signals were observed in the liver, lung, and spleen at 1 h post-injection, and thereafter, the fluorescence signals in all of the organs gradually decreased (Fig. 2b). Intriguingly, at all time points, both the whole body images and the tissue distribution images showed relatively strong fluorescence signals in the spleen and liver (Fig. 2c), which could be explained by the abundant distribution of macrophages strongly expressing mannose receptors in both organs^22^,^23^. This pattern of accumulation is also a common characteristic among medium-sized (10–300 nm in diameter) probes, which typically accumulate in organs containing higher numbers of macrophages^24^. In the additional experiments conducted by comparing the fluorescence in the liver of MMR-Cy5.5 injected and NT-Cy5.5 injected mice (Supplementary Fig. 5c), enhanced fluorescence signals were detected in the MMR-Cy5.5 injected mice as compared to NT-Cy5.5 injected ones, while there were no differences in the degree of mannose receptor expression, suggesting the comparably high specific binding of the MMR-NIRF probe to mannose receptors abundantly expressed in the liver. (Supplementary Fig. 6).

For *in vivo* toxicity studies, major organs such as the kidney, liver, lung, and spleen were excised from mice at 72 hours after intravenous administration of the MMR-Cy5.5 at a dose of 10 mg/kg (n = 3) (Supplementary Fig. 5d). Organ toxicities were investigated under hematoxylin and eosin (H&E) staining. There was no notable evidence of histologic change or damage compared to normal tissue (Fig. 2d). In addition, *in vitro* cytotoxicity of the probe was evaluated in RAW264.7 cells using a CCK-8 kit. After 24 h of incubation with the probe (0–1000 μg/mL), viabilities of RAW264.7 cells were maintained over 95% under the concentration level of 250 μg/mL, and over 85% in between 500 and 1000 μg/mL (Fig. 2e), suggesting that the MMR-targeting NIRF probe was safe without inducing any cellular toxicity.

### *In Vivo* Molecular Imaging of Macrophage-rich Plaques in Atherogenic Mice

**Figure 3:**
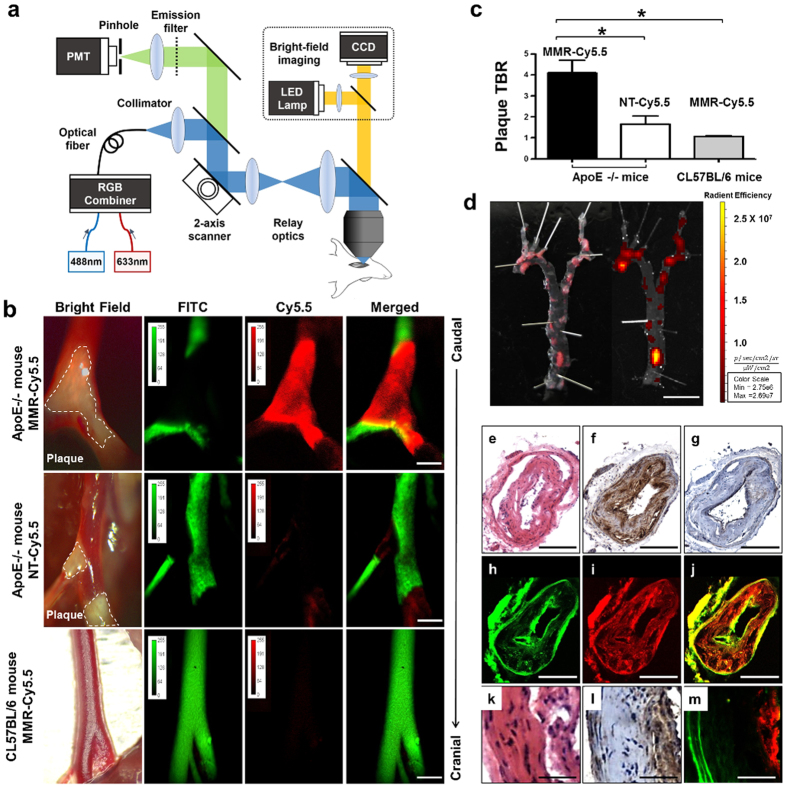
*In Vivo* Optical Imaging of MMR-Cy5.5 in Atherogenic Mice with Histologic Validation. (**a**) Schematic diagram of the custom-built IVFM. (**b**) In all apoE−/− mice, atherosclerotic plaque lesions (dotted white line) were clearly visible in bright-field images. IVFM of the carotid arteries revealed plaque specific high NIRF signals (red) in comparison with the FITC induced angiography (green). Enhanced NIRF signals were only visualized in the MMR-Cy5.5-injected apoE−/− mice. ApoE−/− mice injected with NT-Cy5.5 showed weak NIRF signals demonstrating the superior binding efficacy of MMR-Cy5.5. There were no visible signals in C57BL/6 mice injected with MMR-Cy5.5. Note that a metal plate is placed under the carotid artery, counteracting heart beating and respiratory motion to provide high-resolution images. (**c**) Peak pTBR was significantly greater in MMR-Cy5.5-injected apoE−/− mice than control groups (*p < 0.05). (**d**) Comparison of ORO staining (left panel) and corresponding fluorescence imaging (right panel) of a representative *en face* aorta of MMR-Cy5.5-injected apoE−/− mice. H&E staining (**e**) and Mac3 immunohistochemical staining (**f**) showed macrophages infiltrating the neointima of the carotid artery. Comparing macrophage infiltration, smooth muscle cell proliferation shown by SMA immunohistochemical staining (**g**) was relatively scanty. Autofluorescence signals from the elastin (green) (**h**), fluorescence signals from MMR-Cy5.5 (red) (**i**), and the merged image (**j**) showed good co-localization with the immunohistochemical stainings, corroborating *in vivo* findings. In high magnification, NIRF signals (**m**) showed outstanding co-localization with macrophage infiltration shown in H&E staining (**k**) and Mac3 staining (**i**). Scale bars, 1 mm (**b**), 5 mm (**d**), 100 μm (**e**–**j**), and 25 μm (**k**,**m**).

For *in vivo* molecular imaging, we used a custom-built intravital fluorescence microscopy (IVFM) which is capable of imaging both angiographic and NIRF signals through two different excitation wavelengths (488 nm for fluorescein isothiocyanate [FITC]-dextran and 633 nm for Cy5.5), and obtained bright-field images from the same field of view (Fig. 3a). To optimize the imaging quality, the sample stage could be tilted appropriately to position the carotid artery horizontally, and motion artifacts occurring from pulsation and respiratory movements were minimized by mechanical stabilization, which was achieved by placing a metal plate under the carotid artery (Supplementary Fig. 7). To demonstrate the efficacy and specificity of the MMR-NIRF probe, apoE−/− mice injected with either MMR-Cy5.5 (n = 12) or NT-Cy5.5 (n = 3), and age-matched wild type C57BL/6 mice injected with MMR-Cy5.5 (n = 5) were compared with each other (Supplementary Fig. 8a). 48 hours after intravenously injecting MMR-Cy5.5 into apoE−/− mice, *in vivo* confocal microscopic imaging showed highly enhanced fluorescence signals specifically emitting from carotid plaques identified by the bright-field imaging, while very weak NIRF signals were detected in the NT-Cy5.5-injected group. In contrast, nonspecifically scattered weak background signals were detected in the carotid arteries of MMR-Cy5.5-injected C57BL/6 control mice (Fig. 3b). Three-dimensional rendering of the *in vivo* confocal images obtained vertically up to 150 μm in the carotid arteries of apoE−/− mice injected with MMR-Cy5.5 demonstrated the sufficient penetration depth of this probe into the atheroma, visualizing the plaque volume within the artery (Supplementary Video 1). These results support the specific binding of the MMR-Cy5.5 to plaque macrophages in *in vivo* environments, which is consistent with the *in vitro* studies. The plaque target-to-background ratio (TBR) was significantly higher in MMR-Cy5.5-injected apoE−/− mice compared to the other two groups (vs. NT-Cy5.5-injected apoE−/−; 4.10 ± 0.60 vs. 1.7 ± 0.38, p = 0.03; vs. MMR-Cy5.5-injected C57BL/6; 4.10 ± 0.60 vs. 1.02 ± 0.05, p = 0.003, Fig. 3c, Supplementary Fig. 9). We also examined plaque-specific NIRF signals emitting from the *en face* aorta of MMR-Cy5.5-injected apoE−/− mice using *ex vivo* fluorescence reflectance imaging (FRI). Highly enhanced NIRF signals were observed at plaque lesions across the whole aorta, which well corresponded to atheromata confirmed with Oil Red O (ORO) staining (Fig. 3d).

To determine whether the Cy5.5-fluorescence signals were emitted from plaque macrophages, histopathological validation was performed through H&E (Fig. 3e), immunohistochemical staining with Mac3 (Fig. 3f) and smooth muscle actin (SMA, Fig. 3g). Autofluorescence signals from elastin in the vessel wall were color-coded with green (Fig. 3h), NIRF signals from Cy5.5 were color-coded with red (Fig. 3i), and each channel image was merged (Fig. 3j). While smooth muscle cell proliferation was relatively scarce, abundant infiltration of macrophages was observed in the carotid plaques of apoE−/− mice, and this Mac3-positive area co-localized well with the intense NIRF signals, indicating that the MMR-Cy5.5 specifically targeted at plaque macrophages. Likewise, in the high magnification images of plaque lesions determined by H&E staining (Fig. 3k), highly enhanced NIRF signals co-localized with the dense macrophage contents in the luminal area (Fig. 3l,m), corroborating the *in vivo* imaging results with respect to MMR-Cy5.5 targeting macrophage-abundant atheromata.

### Intravascular OCT-NIRF Imaging of Plaque Inflammation in Coronary-sized Vessels of Atheromatous Rabbits

**Figure 4:**
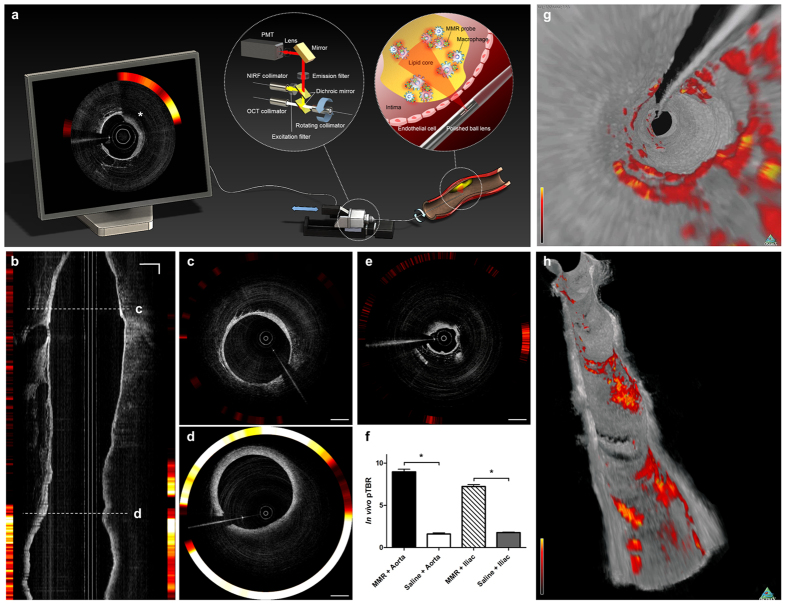
*In Vivo* Imaging of Inflamed High-risk Plaques in Coronary-sized Vessels using MMR-Cy7 and the OCT-NIRF Catheter-Based Imaging System. (**a**) Schematic diagram of the high-speed dual-modal OCT-NIRF catheter-based imaging system. The rotary junction in the center rotates at 100 rps. The ball lens simultaneously collects the OCT and the NIRF signals, which propagate through the double-clad fiber (DCF). The two different signals are separated with the dichroic mirror and are rendered through a custom-built software showing the final OCT-NIRF image presented on the monitor (asterisk), which demonstrates enhanced NIRF signals existing in the macrophage-abundant plaque lesion identified by OCT. (**b**) A longitudinal OCT-NIRF image and (**c**) the corresponding cross-sectional images of a normal lesion, (**d**) an advanced and (**e**) early atherosclerotic plaque lesion. Compared to the normal-looking segment, the NIRF signals emits more prominently from the macrophage-abundant atherosclerotic plaque. (**f**) The *in vivo* pTBR is significantly higher in the MMR-Cy7 injected rabbits compared to the saline-injected control group (*p < 0.05) regardless of the vessel site. (**g,h**) Three-dimensional OCT-NIRF rendered images. (**g**) A fly-through and (**h**) a longitudinal cutaway view enables the identification of both NIRF signals and luminal morphology. Scale bars, 1 mm.

Next, we examined whether the scale-up of this MMR-NIRF probe can be applied for identification of high-risk plaques in coronary-sized arteries. To obtain images of plaque inflammation and the surrounding vascular structure in high resolution simultaneously, we used the fully integrated high-speed dual-modal OCT-NIRF catheter-based imaging system, which was previously developed in our group^15^,^17^,^18^. The simplified underlying design and mechanism of recruiting both OCT and NIRF signals in the OCT-NIRF imaging system is depicted in Fig. 4a. The MMR-targeting nanoparticle was labeled with Cy7 (MMR-Cy7) which has an excitation/emission wavelength of 750/773 nm. Due to its longer wavelength compared to Cy5.5, we could enhance tissue penetration, reduce autofluorescence^25^, and eventually obtain optimal *in vivo* images when combined with the OCT-NIRF catheter. We intravenously injected MMR-Cy7 (10 mg/kg) into balloon-injured rabbits (n = 6) fed with high-cholesterol diet 48 hours prior to *in vivo* imaging (Supplementary Fig. 8b). Intravascular OCT-NIRF imaging was performed in the infrarenal aorta and both iliac arteries at a high pullback speed (20 mm/s) just under contrast flushing (Supplementary Video 2). To aid in the precise quantitation of *in vivo* macrophages in plaque, NIRF signals *in vivo* were calibrated according to the dependence of the NIRF signal intensity on the distance from the catheter tip to a targeted site (Supplementary Fig. 10)^17^. Both longitudinal (Fig. 4b) and cross-sectional (Fig. 4c–e) images obtained from the OCT-NIRF catheter-based imaging system revealed highly enhanced NIRF signals specifically emitted from plaque lesions in the aortoiliac arteries whose distinct morphology was visualized by the OCT. The cross-sectional OCT-NIRF images demonstrated in-depth findings showing stronger NIRF signals in advanced plaque areas (Fig. 4d) compared to relatively weak NIRF signals limited to focal early plaque and none in normal segments (Fig. 4c,e). The OCT-NIRF imaging system could detect NIRF signals even at an administration dose of 1 mg/kg. However, the fluorescence intensity was insufficient for the clear identification of inflamed plaques *in vivo* (Supplementary Fig. 11). A dose of at least 2.5 mg/kg was required to clearly distinguish advanced plaques from early-stage lesions. *In vivo* plaque TBR was measured in each pullbacks obtained from aorta and both iliac arteries. Regardless of the arterial type, pTBR in the MMR-Cy7-injected vessels were significantly higher compared to the saline-injected control group (Aorta; 9.73 ± 0.73 vs. 1.67 ± 0.09, p < 0.05, Iliac; 7.17 ± 0.24 vs. 1.77 ± 0.06, p < 0.001) (Fig. 4f). Three-dimensional fly-through and longitudinal cutaway views (Fig. 4g,h) and a fly-through movie (Supplementary Video 3) demonstrated good co-localization between the intense NIRF signals and the OCT-delineated plaque lesions, providing the axial extent of inflammation and atheromatous stenotic segments.

**Figure 5:**
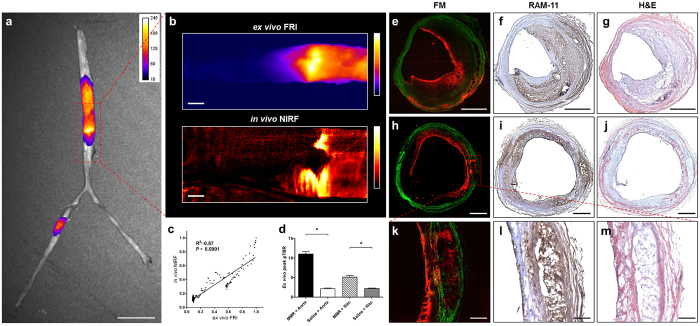
*Ex vivo* Imaging and Histologic Validation of Atheromatous Rabbits. (**a**) *Ex vivo* FRI overlapped with the corresponding white light image. Enhanced NIRF signals are visualized in plaque lesions identified by the white light image. (**b,c**) Co-localization of *ex vivo* FRI NIRF signals with *in vivo* NIRF two-dimensional mapping. (**d**) Higher peak pTBR in the MMR-Cy7-injected arteries compared to that of the saline-injected control group (*p < 0.05). (**e–g**) FM and immunohistochemical staining of an eccentric plaque. The enhanced NIRF signals co-localized well with the RAM-11-stained macrophages. (**h–j**) FM and immunohistochemical staining of a diffuse encircling plaque. Similar results were obtained showing a good co-localization of NIRF signals with macrophage immunostaining in the plaque. Scale bars, 500 μm. (**k–m**) Magnified images of a deep area of within the plaque. Sufficient tissue penetration of MMR-Cy7 was identified in the atheroma with abundant macrophage accumulation. Scale bars, 10 mm (**a**), 3 mm (**b**), 500 μm (**e–j**), 200 μm (**k–m**).

After carefully removing the aorta and both iliac arteries, *ex vivo* FRI was performed for validation of *in vivo* NIRF imaging. *Ex vivo* FRI revealed strong NIRF signals within plaque lesions which were induced by balloon denudation and high-cholesterol diet (Fig. 5a). FRI of a vessel containing plaque and normal segment corresponded well to two-dimensional *in vivo* NIRF signal mapping (Fig. 5b). The *in vivo* NIRF signals significantly co-localized with the *ex vivo* FRI signals (R^2^ = 0.87, p < 0.0001, Fig. 5c). Plaque TBRs obtained from *ex vivo* FRI images were much higher in the MMR-Cy7-injected rabbits as compared to those in the saline-injected control group (n = 3), with a 5-fold increase in the aorta (11.01 ± 0.63 vs. 2.16 ± 0.17, p < 0.05) and a 3-fold in the iliac arteries (5.13 ± 0.44 vs. 2.21 ± 0.12, p < 0.05, Fig. 5d).

For histopathological validation, fluorescence microscopy (FM) images, RAM-11 immunohistochemical staining for macrophage contents, and H&E staining were performed in sister sections. Highly enhanced NIRF signals (Fig. 5e,h) in advanced plaque lesions (Fig. 5g,j) showed good co-localization with macrophages in the plaque (Fig. 5f,i). The MMR-Cy7 probe demonstrated sufficient tissue penetration in macrophage-abundant plaque portions, which was nicely shown by high-magnification microscopic images (Fig. 5k,m, Supplementary Fig. 12a–c). Focal atheroma in the early stage showed weak NIRF signals compared to advanced lesions, but still demonstrated good co-localization with macrophages recruited within the cap (Supplementary Fig. 12d–f).

## Discussion

We report the first optical molecular imaging of high-risk atherosclerotic plaques using a novel, MMR-targeting NIRF probe *in vivo*. With the custom-built IVFM system, we demonstrated this probe was feasible for identifying *in vivo* macrophage-rich atheromata in small animals such as carotid plaques in apoE−/− mice. As combined with our intravascular high-speed dual-modal OCT-NIRF catheter-based imaging system, the scale-up of our MMR-targeting NIRF probe could be successfully applied for identification of high-risk plaques in coronary-sized arteries *in vivo*. Moreover, this MMR-targeting NIRF probe proved to be safe by *in vitro* and *in vivo* validation.

During the last few decades, pre-clinical cardiovascular imaging studies for atherosclerotic plaques using nanoprobes labeled with various imaging agents have been implementing to detect inflamed high-risk plaques^26^. The plaque targeting mechanism of previous nanoprobes relied on non-specific phagocytosis by local macrophages after paracellular extravasation through the disrupted endothelium or leakage from vasa vasorum and neovessels^27^. In comparison with these relatively passive courses, our MMR-targeting NIRF probe enables active targeting of mannose receptors which has an endocytic function, thus facilitating more specific and effective cellular internalization.

Another important benefit of targeting mannose receptors is that the receptors are associated with high-risk plaque phenotypes such as TCFA^19^, neovascularization, and intraplaque hemorrhage^19^,^20^,^21^. Recently, positron emission tomography (PET) imaging with 2-deoxy-2-[^19^F]fluoro-D-mannose was developed for high-risk plaque imaging. However, critical for the non-invasive PET molecular imaging is the limited resolution of the platform for tiny coronary plaques in a continuous beating heart. Furthermore, without the offline co-registration of another structural imaging, PET imaging could not provide information regarding plaque morphology. To address these issues, we newly developed a NIRF-conjugated optical imaging probe targeting MMR, based on glycol chitosan. This naturally existing polysaccharide is a non-toxic, biocompatible, biodegradable, and poorly immunogenic biopolymer^28^, which has been thoroughly investigated as an *in vivo* tumor imaging agent with a high degree of safety^29^,^30^,^31^,^32^. The attachment of PEG polymer chains to the MMR-targeting probes has provided additional advantages regarding increased stability and circulation time in *in vivo* circumstances by reducing clearance through the reticuloendothelial system^33^.

After we confirmed the feasibility of this MMR-targeting NIRF probe to detect plaque inflammation *in vivo* in an atheromatous mouse model, we applied the MMR-NIRF probe for coronary-sized inflamed plaques by catheter-based OCT-NIRF imaging. Our group has recently developed the intravascular dual-modal high-speed OCT-NIRF catheter-based imaging system to simultaneously image plaque morphology and molecular pathways in coronary-sized arteries^17^,^18^. Herein, with this novel dual-modal catheter-based imaging, we adapted the MMR-targeting NIRF probe to quantitatively image plaque inflammation in coronary-sized arteries. Although activatable NIRF sensors targeting inflammation efficiently produce fluorescence signal amplification, NIRF signals were inevitably exaggerated through the catalytic properties through enzymes such as cysteine proteases^11^,^12^,^34^,^35^. In contrast, the specific binding of our NIRF probe to MMRs enables direct estimation of plaque macrophage contents. As NIRF signal intensity attenuates depending on the distance from catheter imaging tip to the target site, we calibrated NIRF data according to the dependence of the NIRF signal intensity on the distance. OCT-NIRF imaging with the MMR-targeting NIRF probe accordingly has benefit in quantitative assessment of coronary plaque risk more accurate than any other imaging strategies. Of note, our *in vitro* and *in vivo* toxicity studies demonstrated promising results regarding the safety of this agent. While further validations including long-term safety should be required, based on current data, the MMR-targeting NIRF probe represents a promising optical imaging agent with high potential for clinical translation.

The clinical implications of our novel strategy pertain to the prediction of coronary events risk. Based on all findings in this research, the high-speed intravascular OCT-NIRF catheter-based imaging with our MMR-targeting NIRF probe offers considerable promise for intracoronary detection of vulnerable plaques in human arteries. By providing complementary information regarding both plaque morphology and macrophage subsets with respect to high-risk atheroma phenotypes, this dual-modal structural-molecular imaging strategy is expected to surmount the limitations of conventional coronary imaging. The potency of this imaging approach addresses the unmet need for developing a more accurate diagnostic strategy for high-risk coronary plaques, and is expected to offer a new avenue for personalized approaches to predict coronary events.

## Methods

### Nanoparticle Synthesis and Fluorescence Labeling

#### Synthesis of MAN-PEG-MAL

Maleimide-PEG2000-NHS ester (300 mg, JENKEM), mannosamine hydrochloride (48.5 mg, Sigma) and triethylamine (TEA, 47.3 μL, Sigma) were dissolved in 8 mL of anhydrous dimethylformamide (DMF, Sigma). After overnight reaction, the mixtures were dialyzed against distilled water using a dialysis membrane (molecular weight cut-off (MWCO): 1,000, Spectrum) for 48 h and lyophilized (Supplementary Fig. 1).

#### Synthesis of MMR-targeting Probe

The synthetic scheme is shown in Supplementary Fig. 2. To synthesize the NIRF optical molecular imaging probe capable of targeting MMR, first, 500 mg of glycol chitosan (GC, Mw = 250 kDa) dissolved in 4-morpholineethanesulfonic acid sodium salt buffer (pH 5.6) was reacted with N-acetylcysteine (16.3 mg) in the presence of N-hydroxysuccinimide (NHS, 17.3 mg) and 1-ethyl-3-(3-dimethylaminopropyl)carbodiimide hydrochloride (28.7 mg). After reaction for 48 h, the mixtures were dialyzed against distilled water using a dialysis membrane (MWCO 6,000–8,000 Da), and lyophilized to obtain thiolated GC (tGC). To endow targetability to mannose receptors, MAN-PEG-MAL (112 mg) was reacted with tGC (400 mg) in PBS (70 mL, pH 6.9) for 20 h, dialyzed against distilled water using a dialysis membrane (MWCO 12,000–14,000 Da) for 2 days, and lyophilized to obtain MAN-PEG-GC. For the NT nanoprobe, methoxy PEG (mPEG)-MAL (108 mg) was reacted with tGC (400 mg) under the same conditions. To fabricate self-assembled nanostructures and to aid in visualizing the plaques, MAN-PEG-GC (100 mg) or mPEG-GC (100 mg) was conjugated with cholesteryl chloroformate (5 mg) in 20 mL of DMSO:DMF (3:1, v/v) containing TEA (9 μL) for 24 h, and further reacted with 2 mg of Cy5.5-NHS ester (or Cy7-NHS ester, Lumiprobe) in darkness overnight. Then, the mixture was dialyzed against distilled water: EtOH (1:2, v/v) for 2 days using a dialysis membrane (MWCO 12,000–14,000 Da) and further dialyzed against distilled water for additional 2 days to remove unreacted cholesteryl chloroformate and Cy5.5 (or Cy7), and freeze-dried to finally obtain MMR-targeting probes or NT probes labeled with Cy5.5 or Cy7.

#### *In Vitro* Cellular Uptake and Cellular Binding Study

The RAW264.7 cells (1 × 10^5^ cells) were seeded onto glass-bottom dishes for 2 days. To evaluate time-dependent cellular uptake of MMR-Cy5.5 in macrophage cells, RAW264.7 cells were replaced with fresh media containing MMR-Cy5.5 (200 μ μg/mL) and incubated at 37 **°**C for 10 min, 30 min, 1 h, and 2 h. To further investigate dose-dependent cellular uptake, RAW264.7 cells were treated with various concentrations of MMR-Cy5.5 (0–200 μg/mL) for 1 h. Then, the cells were washed twice with ice-cold PBS (pH 7.0), fixed with fresh 4% paraformaldehyde for 5 min at room temperature, and treated with CellMask^TM^ Orange (2.5 μg/mL) at 37 **°**C following the supplier’s protocol to visualize the plasma membrane. The nuclei of the cells were counterstained with 4′,6′-diamidino-2-phenylindole hydrochloride (DAPI)-Fluoromount-G^TM^ (SouthernBiotech, Birmingham, AL).

To further evaluate whether MMR-Cy5.5 specifically binds with MRs expressed on macrophages, RAW264.7 cells (1 × 10^5^ cells/well) were maintained with fresh media containing MMR-Cy5.5 (100 μg/mL) or NT-Cy5.5 (100 μg/mL) and incubated at 37 **°**C for 1 hr. For blocking experiments, free mannosamine (500 μM) was pre-incubated for 1 h, and the cells were then further maintained with fresh media containing MMR-Cy5.5 (100 μg/mL) for 1 h. The cells were washed and fixed with fresh 4% paraformaldehyde, and the nuclei of the cells were counterstained with DAPI. The intracellular uptake and cellular binding studies were visualized under a laser scanning confocal microscope (LSM 780 Meta, Carl Zeiss, Thornwood, NY).

#### *In Vitro* Cell Viability Assay

*In vitro* cytotoxicity of the MMR-Cy5.5 against RAW264.7 was evaluated using a CCK-8 assay kit. RAW264.7 cells (5 × 10^4^ cells) were seeded in 96-well plates and allowed to adhere for 24 h. The cells were washed twice with culture medium and incubated with various concentrations of the MMR-Cy5.5 (0, 10, 25, 50, 100, 250, 500, and 1000, or 0–1000 μg/mL). After 24 h of incubation, the cells were washed twice with culture medium. According to the manufacturer’s protocol, we performed the CCK-8 assay to determine cell viability, and the absorbance was measured at 450 nm. Untreated cells were used as the control group (100% viability). Relative cell viability (%) was calculated by the following equation: [abs]_test_/[abs]_control_ × 100.

#### Biodistribution and *In Vivo* Toxicity

The protocols of experiments for evaluating biodistribution and toxicity are shown in Supplementary Fig. 5. To evaluate the time-dependent excretion profile, normal C57BL/6 nude mice (n = 5) were injected with MMR-Cy5.5 at 10 mg/kg via the lateral tail vein. NIRF whole body images were monitored and obtained using a small animal optical imager (IVIS 200, Perkin Elmer) at 0, 1, 2, 3, 6, 12, 24, 48, 72, and 120 h post-injection. In addition, to investigate tissue distribution, the same dose of MMR-Cy5.5 was intravenously injected into wild type C57BL/6 mice (n = 3 per time interval). The major organs such as the liver, lung, kidney, spleen, and heart were excised and imaged with IVIS 200 at 1, 3, 6, 12, 24, 48, and 72 h post-injection, and the fluorescence signals per 1 g of tissue were determined. To verify the origin of the relatively strong fluorescence signals in the liver, NIRF signals emitting from the liver were compared by *ex vivo* FRI (INV-16 M, Davinch-K Co., Seoul, Korea), FM, and immunohistochemical staining with CD206 (MCA2235GA, AbD Serotec, Oxford, UK) after administrating the same dose (10 mg/kg) of NT-Cy5.5 or MMR-Cy5.5 to C57BL/6 mice (n = 3 for each group, Supplementary Fig. 5c). Additionally, to evaluate the *in vivo* toxicity of the administration of MMR-Cy5.5, histopathological evaluation of the excised tissues including the kidney, liver, lung, and spleen from wild type C57BL/6 at 72 h post-injection was performed. The excised tissues were fixed in 10% formalin, paraffin-embedded, and stained with H&E according to standard protocols. An experienced pathologist analyzed each tissue.

#### Multichannel Laser Confocal IVFM System

We developed an IVFM system to acquire comparative images of bright-field, angiogram (FITC) and plaque specific NIRF signals from live mouse carotid arteries. To increase accessibility to mouse carotid arteries *in vivo*, we adopted an upright epi-fluorescence illumination laser confocal configuration with a sample stage with 5 degrees of freedom^36^. A bright-field image of the carotid artery was captured to observe the gross structure of the carotid artery using a white-light illuminator (LIU104, Thorlabs) and a CCD (DCU223C, Thorlabs) having the same field of view as the fluorescence images. FITC and Cy5.5 were excited using 488 nm and 633 nm lasers, respectively. The fluorescent emission signals were detected with a photomultiplier tube (H7422P-40, Hamamatsu Photonics) through a multi-band dichroic beam splitter (Di01-R488/543/635, Semrock), a multi-band emission filter (FF01-515/588/700, Semrock) and a variable confocal pinhole (MPH16, Thorlabs). High-speed imaging up to 7 frames per second at 1024 × 512 pixels was achieved by using a resonant scanning mirror (CRS4 kHz, Cambridge Technology). Fast image acquisition minimized motion blur due to pulsation, enabling the visualization of the exact plaque shape. Using a 10 × /0.30 NA objective lens, a large imaging field of view (up to 2 × 2 mm) was acquired with lateral and axial resolution of 1.0 μm and 15 μm, respectively. Axial scanning was performed using a piezoelectric objective positioner (MIPOS 500 SG, Piezosystem jena).

#### Advanced High-speed Dual-modal OCT-NIRF Catheter-Based Imaging System

We previously developed a fully-integrated high-speed OCT-NIRF catheter-based imaging system^17^,^18^. A schematic diagram of the advanced, intravascular dual-modal OCT-NIRF system is presented in Fig. 4a. The two different modalities were integrated through a dual-modal fiber-optic rotary junction (FRJ). A collimator using the double-clad fiber (DCF) was custom-built to form the rotary part of the FRJ. Both the OCT imaging light and the NIRF excitation light coupled to the core of the DCF collimator and then to the core of the DCF-based imaging catheter. The two lights were focused on the target of interest by the ball lens at the distal tip of the imaging catheter, which was placed inside the vessel. OCT and NIRF signals reflected from the target transmitted back to the core and cladding of the DCF imaging catheter, respectively. The FRJ rotated the catheter at either 50 or 100 revolutions per second during the procedure. The OCT and NIRF signals were separated with a dichroic mirror and directed to the OCT interferometer and photo multiplier tube (PMT), respectively. The detected signals were digitized and processed, and the real-time OCT-NIRF images were presented on the system monitor through the custom-built real-time visualization software.

#### Animal Preparation and *In Vivo* Optical Imaging

All of the animal experiments were approved by the Institutional Animal Care and Use Committee of Korea University (KUIACUC-2014-169), and all animal procedures were performed in accordance with the relevant guideline. Detailed experimental protocols for the *in vivo* imaging studies of mice and rabbits are illustrated each in Supplementary Fig. 8a,b.

#### *In vivo* Imaging of ApoE−/− Mice

Anesthesia was administered before surgical procedures and *in vivo* imaging studies (intraperitoneal injection of zoletil 30 mg/kg and Rompun 10 mg/kg). The animals were monitored regularly for pain or discomfort. Ten–week-old male apoE−/− mice (n = 12) and age-matched C57BL/6 mice (n = 5) were obtained from Japan SLC, Inc. (Hamamatsu, Japan), and fed with a high cholesterol diet containing 0.25% of cholesterol for 20 weeks. Forty-eight hours before imaging, MMR-Cy5.5 (10 mg/kg) was injected into the apoE−/− mice and the C57BL/6 mice via the tail vein. NT-Cy5.5 was injected into the apoE−/− mice (n = 3) using the same dose and injection route. Before the imaging studies, FITC-dextran (10 mg/kg, MW 2,000,000) was injected via the tail vein as an angiographic fluorescent imaging agent. The left carotid artery of the mice was surgically exposed, and a metal plate derived from palate knife was placed underneath the carotid artery. Using the metal plate, which did not emit any fluorescence signals, the carotid artery was raised and fixed to minimize signal disturbance from the surrounding tissue and to prevent signal blurring due to pulsation. First, bright-field images were acquired to observe the plaque structure and determine the field of view. Then, *in vivo* florescence imaging was performed using our customized IVFM with an excitation wavelength of 633 nm for Cy5.5, and 488 nm for FITC. The platform supporting the imaging subject was tilted to establish an optimal angle for obtaining a clear view of the carotid plaque. After *in vivo* imaging, the mice were terminated and the aorta and carotid arteries were excised *en bloc* followed by histologic analysis.

#### *In vivo* Imaging of High-risk Plaques in Coronary-sized Vessels of Atheromatous Rabbits

Atherosclerotic lesions were induced in New Zealand white rabbits (n = 9, NZWR, Charles River Laboratories, Wilmington, MA) with balloon denudation and high cholesterol diet (1% cholesterol and 5% peanut oil, C-30293, Research Diets). Balloon denudation was performed at the infrarenal aorta and right iliac artery with a total of three consecutive pullbacks at a balloon pressure ranging from 0.05 to 0.2 mL depending on the site of injury. Intravascular imaging of the inflamed plaque lesions was performed at least 8 weeks after balloon injury. 48 hours prior to *in vivo* imaging, we intravenously injected MMR-Cy7 at a dose of 10 mg/kg. On the imaging day, the left carotid artery was carefully exposed followed by insertion of a 5-F sheath catheter under anesthesia (intramuscular ketamine (50 mg/kg) and xylazine (5 mg/kg)). Standard 0.014-inch guide wire was employed after baseline angiography under fluoroscopic guidance. The guide wire was placed in the previously injured iliac artery. The advanced intravascular dual-modal OCT-NIRF catheter with an automated rotational pullback system (Fig. 4a) was then inserted. We performed three consecutive pullbacks each at the balloon-injured right iliac artery and infrarenal aorta (rotational speed, 100 rps; pullback speed, 20 mm/s), using the same anatomical landmarks under contrast flushing through the catheter. To evaluate the minimum dose of MMR-Cy7 needed for *in vivo* detection by the OCT-NIRF catheter, we further performed *in vivo* imaging after injecting MMR-Cy7 at the dosages of 1, 2.5, and 7.5 mg/kg. After acquiring the images *in vivo*, rabbits were euthanized for full dissection of both iliac arteries up to the descending aorta to perform *ex vivo* imaging.

### Image Analysis

The images were analyzed with the public-domain ImageJ software (version 1.47, US National Institutes of Health, Bethesda, MD). Fluorescence signal intensity was quantitatively analyzed by placing a region of interest (ROI) on atherosclerotic plaque lesions developed in both of the animal models. The ROI was manually traced within the plaque and the normal carotid artery. The plaque TBR was defined as: (ROI values from the atherosclerotic plaque)/(ROI values from the normal carotid artery) (Supplementary Fig. 9). Each image was windowed and processed identically before analysis. 3D rendering was done using a DICOM viewer, OsiriX (The OsiriX Foundation, Geneva, Switzerland).

### Histology, Immunohistochemistry, and Confocal Fluorescence Microscopy

#### *Ex Vivo* Imaging and Histological Validation of Atherogenic Mice

After euthanasia, mice were perfused with 0.9% saline via the left ventricle. Aortas were excised from the aortic arch to the renal arteries. Connective tissue and perivascular fat were removed from the excised aortas, and they were then cut longitudinally and flat-mounted *en face* lumen-side up on black boards. *En face* aortas were stained with ORO (Sigma-Aldrich, St. Louis, MO, USA) to confirm the lipid accumulation, and NIRF images were obtained from the vessel using the IVIS 200 (Perkin Elmer). Carotid arteries were also dissected carefully and cut into two 3 mm sections. Then the dissected carotid arteries were embedded in optimal cutting temperature (OCT, Sakura Finetek, Torrance) compound and rapidly frozen using liquid nitrogen. The frozen tissues were sliced in 7 μm thick sections ready for immunohistochemical staining and 20 μm thick sections for confocal FM. To visualize macrophages and smooth muscle cells in the atherosclerotic plaque, sections were stained with anti-mouse Mac3 (BD biosciences Pharmingen, San Diego, CA), a macrophage marker, and SMA, an anti-mouse alpha SMA antibody (Abcam Inc., Cambridge, MA), according to manufacturer’s instruction. Sections were counterstained with H&E. The distribution of MMR-Cy5.5 in plaques was imagined in cryosections by our customized-laser confocal FM (see in Multichannel Laser Confocal IVFM System section above). Cellular structures were observed using a 20× objective lens (0.46 NA). The MMR-Cy5.5 signal was acquired using a 633 nm laser, and autofluorescence of elastin was acquired using a 488 nm laser. ImageJ software was used to quantify the co-localization between MMR-Cy5.5 and Mac3 staining.

#### *Ex Vivo* FRI and Immunohistological Validation of Atheromatous Rabbits

After euthanasia and saline perfusion, *ex vivo* imaging of the excised descending aorta and both iliac arteries was performed with customized-fluorescence reflectance imaging (FRI) consisting of a 300-W Xenon arc lamp (MAX-302; Asahi Spectra Co, Ltd, Japan) and a cooled charge-coupled device camera (PIXIS 1024BR, Roper Scientific). After FRI, tissue blocks of the entire vessel were cut to a length of 3 mm, embedded in OCT compound, and stored at −80 **°**C. Eight to ten thin (10 μm) serial frozen sections were obtained for immunohistochemical staining and two thick (30 μm) sections were obtained for FM. The cryosections were imagined by fluorescence microscopy using a Nikon Eclipse TE2000-U fluorescence microscope (Nikon Instruments, Melville, NY) with filter sets for Cy7-NIRF (excitation/emission: 743/767 nm; exposure time: 10 s) and autofluorescence (excitation/emission: 495/519 nm; exposure time 500 ms). Windowing of images was identical and all images were captured with a CCD camera (ProgRes MF cool, JENOPTIK, Germany) and analyzed using ImageJ software (version 1.47, US National Institutes of Health, Bethesda, MD).

For histopathological analysis, immunohistochemical staining was performed with a monoclonal mouse anti-rabbit antibody (RAM-11, DAKO Corp., 1:500) to identify the macrophage content, and H&E to investigate the overall morphology of the plaque. Histological images were acquired with a CCD camera (PROSILICA EC 655, Allied Vision Tech., Canada) using an Eclipse TE2000-U microscope (Nikon instruments, Melville, NY).

### Statistical Analysis

Data are expressed as mean ± s.e.m. Comparisons of the fluorescence signal intensities between atherosclerotic plaques and normal arteries were analyzed with the nonparametric Mann-Whitney *U* test. Linear regression analysis was performed. All statistical analysis was performed using GraphPad Prism software (GraphPad Software, San Diego, CA, USA). A p value < 0.05 was considered statistically significant.

## Additional Information

**How to cite this article**: Kim, J. B. *et al.* Intravascular optical imaging of high-risk plaques *in vivo* by targeting macrophage mannose receptors. *Sci. Rep.* **6**, 22608; doi: 10.1038/srep22608 (2016).

## Supplementary Material

Supplementary Video 1

Supplementary Video 2

Supplementary Video 3

Supplementary Information
