# Supplementary material for: Intravascular optical imaging of high-risk plaques in vivo by targeting macrophage mannose receptors
Source: Sci Rep. 2016 Mar 7;6:22608. doi: 10.1038/srep22608 (PMC4780083; doi:10.1038/srep22608)
Supplement: Supplementary Information [file srep22608-s4.doc]

**SUPPLEMENTARY INFORMATION**

**Intravascular optical imaging of high-risk plaques *in vivo***

**by targeting macrophage mannose receptors**

Ji Bak Kim^1†^, Kyeongsoon Park^2†^, Jiheun Ryu^3†^, Jae Joong Lee^1†^, Min Woo Lee^4^, Han Saem Cho^3^, Hyeong Soo Nam^4^, Ok Kyu Park^2^, Joon Woo Song^1^, Dong Joo Oh^1^, DaeGab Gweon^3^,
Wang-Yuhl Oh^3*^, Hongki Yoo^4*^, Jin Won Kim^1*^

^1^Multimodal Imaging and Theranostic Lab, Cardiovascular Center, Korea University Guro Hospital, Seoul, Republic of Korea

^2^Division of Bio-imaging, Chuncheon Center, Korea Basic Science Institute, Republic of Korea

^3^Department of Mechanical Engineering, KAIST, Daejeon, Republic of Korea

^4^Department of Biomedical Engineering, Hanyang University, Seoul, Republic of Korea

^†^These authors contributed equally to this work.

^*^These authors shared senior authorship.

**Running title**

*In Vivo* Optical Molecular Imaging of Inflamed Atheromata

**Corresponding Authors**

**Jin Won Kim, M.D., Ph.D.**

Address: Cardiovascular Center, Korea University Guro Hospital, 80, Guro-dong, Guro-gu, Seoul 152-703, Republic of Korea

Tel: 82-2-2626-3021, Fax: 82-2-863-1109, E-mail: [kjwmm@korea.ac.kr](mailto:kjwmm@korea.ac.kr)

**Wang-Yuhl Oh, Ph.D.**

Address: Department of Mechanical Engineering, KAIST, 291 Daehak-ro, Yuseong-gu, Daejeon 305-701, Republic of Korea

Tel: 82-42-350-3237, Fax: 82-42-350-5237, E-mail: woh1@kaist.ac.kr

**Hongki Yoo, Ph.D.**

Address: Department of Biomedical Engineering, Hanyang University, 222 Wangsimni-ro, Seongdong-gu, Seoul 133-791, Republic of Korea

Tel: 82-2-2220-2323, Fax: 82-2-2220-5943, E-mail: hyoo@hanyang.ac.kr

**SUPPLEMENTARY MATERIALS AND METHODS**

**Materials**

Maleimide-PEG2000-NHS ester (MAL-PEG-NHS) and methoxy PEG2000-NHS ester (mPEG-NHS) were purchased from JENKEM Co. Mannosamine hydrochloride (MAN), triethylamine (TEA), anhydrous dimethylformamide (DMF), anhydrous dimethylsulfoxide (DMSO), 4-morpholineethanesulfonic acid sodium salt (MES), glycol chitosan, N-acetyl-L-cysteine (NAC), N-hydroxysuccinimide (NHS), 1-ethyl-3-(3-dimethylaminopropyl)-carbodiimide hydrochloride (EDC), cholesteryl chloroformate, CDCl_3_, D_2_O and CD_3_OD were purchased from Sigma-Aldrich Chemical Co. The near-infrared fluorophores, Cy5.5-NHS ester (Ex: 673 nm, Em: 707 nm) and Cy7-NHS ester (Ex: 750 nm, Em: 773 nm) were purchased from Lumiprobe Co. Dialysis membranes (MWCO: 1 kDa, 6–8 kDa, and 12–14 kDa) were purchased from Spectrum. For the staining of cell nuclei, 4',6'-diamidino-2-phenylindole hydrochloride (DAPI)-Fluoromount-GTM was obtained from SouthernBiotech Co.

**Cells and Animals**

RAW 264.7 cells were obtained from the Korean Cell Line Bank (KCLB, Seoul, Korea). RAW 264.7 cells were cultured in Dubecco’s modified Eagle’s medium (DMEM) containing 10% fetal bovine serum (Hyclone, Logan, UT, USA), 5 mM l-glutamine, and 5 μg/mL gentamicin at 37°C using a humidified 5% CO_2_ incubator.

Wild type C57BL/6 (7 or 10 week-old, male), C57BL/6 nude mice (7 week-old, male), and apoE-/- mice were purchased from Japan SLC, Inc. (Hamamatsu, Japan). During *in vivo* whole body imaging, mice were anesthetized with isoflurane (1%, w/v, JW-Pharma, Korea) in 2 L oxygen. ApoE-/- mice were fed a Western diet containing 0.25% cholesterol for 20 weeks. New Zealand white rabbits (weight, 3.5-4 kg; Charles River Laboratories, Wilmington, MA) were placed on a high-cholesterol diet (1% cholesterol and 5% peanut oil, C-30293, Research Diets) for 1 week before balloon injury. Rabbits were continuously anesthetized with intramuscular ketamine (50 mg/kg) and xylazine (5 mg/kg) during the balloon injury procedure. The handling and care of the animals confirmed to current international laws and policies (NIH Guide for the Care and Use of Laboratory Animals, NIH Publication No. 85-23, 1985, revised 1996) and were approved by the Institutional Animal Care and Use Committee (IACUC) of the Korea University (Protocol No: 3120000) and Korea Basic Science Institute (KBSI-AEC 1303).

**Characterizations of MAN-PEG-MAL**

The synthesized MAL-PEG-MAL was characterized with a MALDI-TOF mass spectrometer (Voyager-DETM STR Biospectrometry Workstatin, Applied Biosystems, Carlsbad, CA) and an ^1^H-NMR spectroscope (Bruker DPX 400 MHz, Germany). The determined molecular weight of MAN-PEG-MAL: m/z = approximately 2,479.3 Da. The chemical structure of MAN-PEG-Mal was confirmed with ^1^H NMR. ^1^H NMR (solvent: CDCl_3_) peaks are as the following: δ 6.7(H of = CH of MAL), δ 3.81-4.16 (H from MAN), δ 3.58-3.74 (H of –CH_2_CH_2_O– from PEG), and δ 2.52 (H of MAL-CH_2_-CH_2_-CO-PEG).

**Characterizations of probes**

The synthesized mPEG-GC-Chol and MAN-PEG-GC-Chol nanoparticles were characterized with ^1^H NMR (Bruker, Germany). ^1^H NMR (solvent: CD_3_OD:D_2_O = 3:1, v/v) peaks are as follows: δ 0.88-02.97(H of CH_3_ from Chol), δ 1.27-1.47 (H of CH or CH_2_ from Chol), and δ 3.59-3.70 (H of –CH_2_CH_2_O– from glycol chitosan and PEG). Particle size and distribution of Cy5.5- or Cy7- labeled MAN-PEG-GC-Chol (called MMR-Cy5.5 or MMR-Cy7 probe) and Cy5.5-labeled mPEG-GC(Cy5.5)-Chol nanoparticles (Called NT-Cy5.5 probe) were characterized with variable pressure field emission scanning microscope (VP-FE-SEM, SUPRA-55-VP, Carl Zeiss, Germany) and/or energy filtering transmission electron microscope (EF-TEM, LEO 912AB OMEGA, Carl Zeiss). No big differences in particle sizes and distributions of MMR-Cy5.5, MMR-Cy7, and NT-Cy5.5 were observed. The degree of substitution of Cy5.5 (or Cy7) per molecule of MAN-PEG-GC-Chol and mPEG-GC-Chol was determined by measuring the extinction coefficient of Cy5.5 at 673 nm (2.09 × 10^5^ M^-1^cm^-1^) (or Cy7 at 750 nm, 1.99 × 10^5^ M^-1^cm^-1^) as described by the manufacturer. On average, each molecule of MAN-PEG-GC-Chol contained 6.7 ± 0.5 molecules of Cy5.5 and 5.8 ± 0.8 molecules of Cy7, respectively. In addition, each molecule of mPEG-PEG-GC-Chol contained 6.4 ± 0.9 molecules of Cy5.5.

**Supplementary Fig 1.** Synthesis of mannosamine-conjugated PEG-maleimide (MAN-PEG-MAL).

**Supplementary Fig 2.** A simplified synthetic scheme of MAN-PEG-GC(Cy5.5 or Cy7)-Chol particles (MMR-Cy5.5 or MMR-Cy7). NIRF-emitting probe capable of targeting macrophage mannose receptor on macrophages.


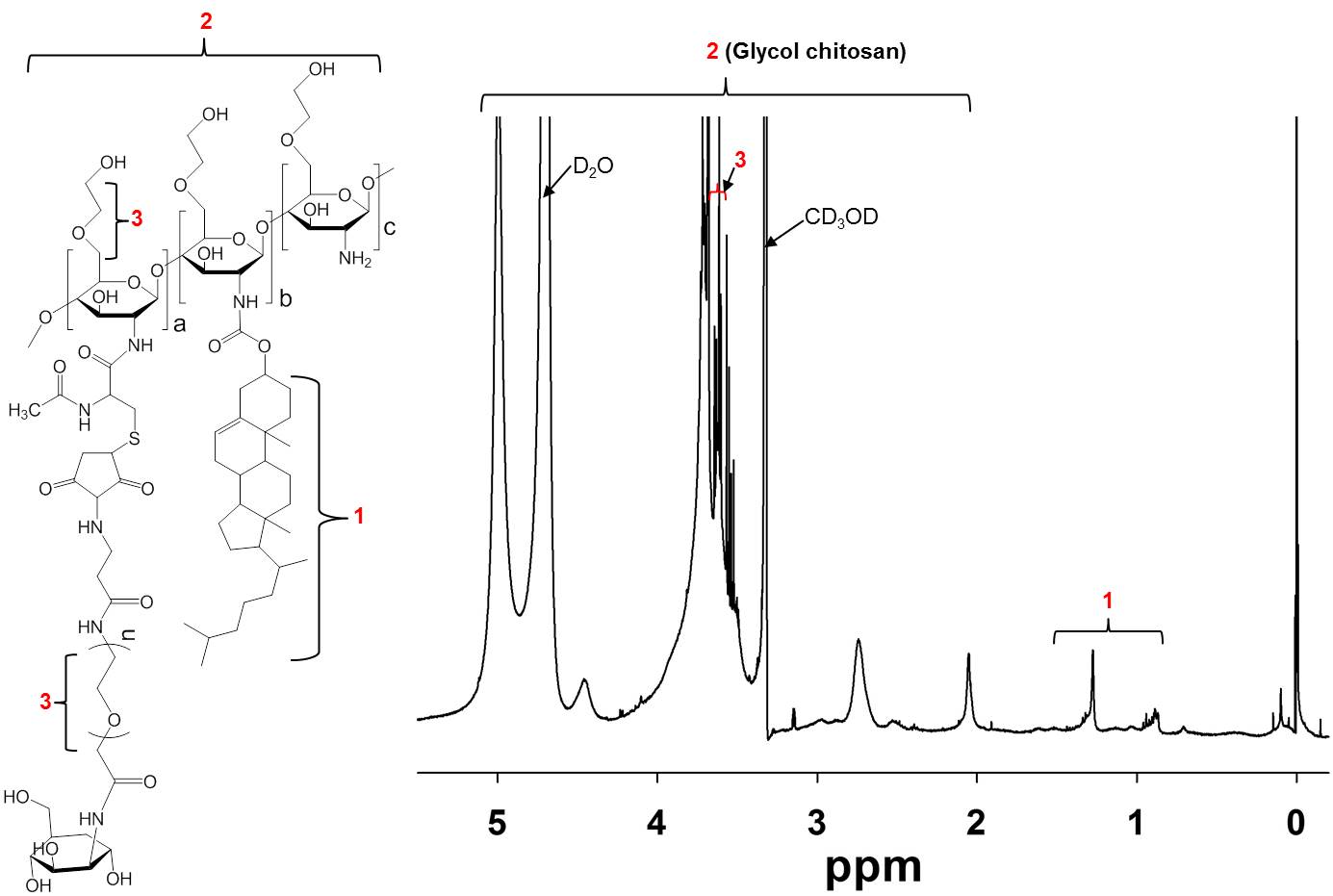


**Supplementary Fig 3.** ^1^H NMR peaks of MAN-PEG-GC-Chol.


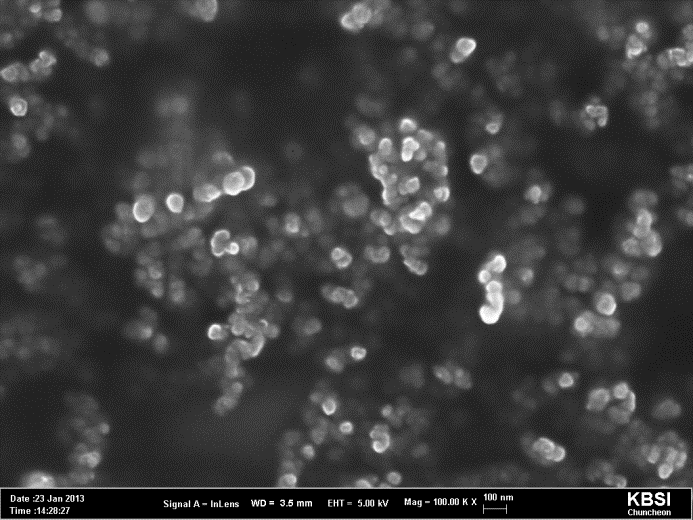

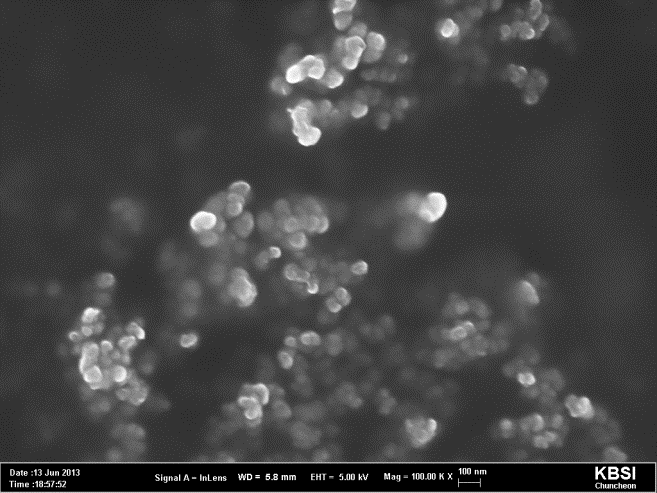

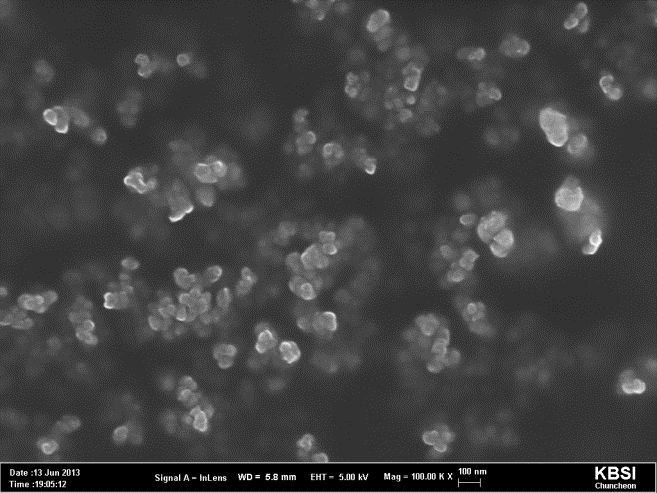


**a**

**b**

**c**

**Supplementary Fig 4**. SEM images of (a) mPEG-GC(Cy5.5)-Chol, (b) MAN-PEG-GC(Cy5.5)-Chol, and (c) MAN-PEG-GC(Cy7)-Chol. Scale bar, 100 nm.


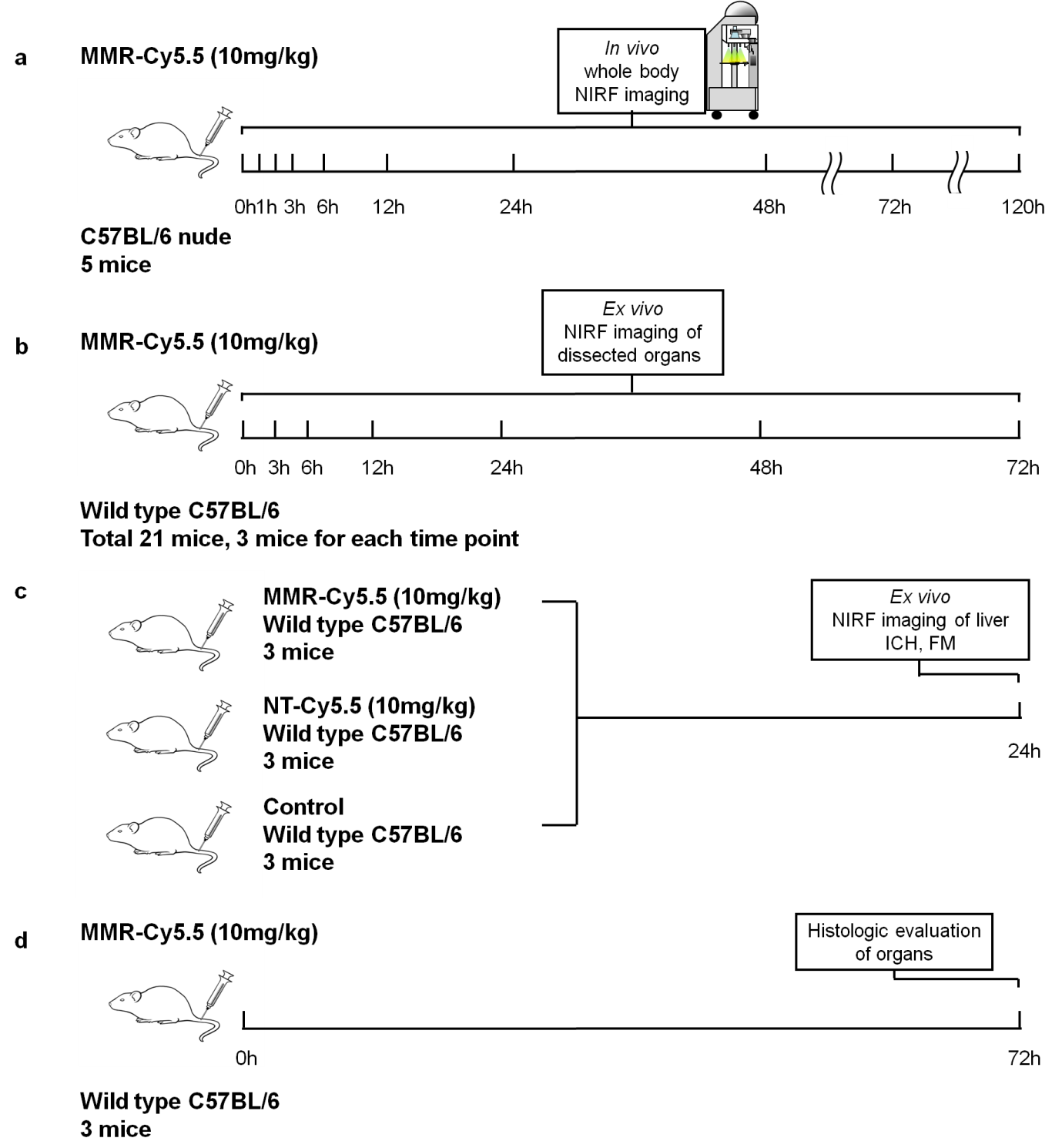


**Supplementary Fig 5. Biodistribution and Toxicity Protocol**

Experimental protocols for the biodistribution and toxicity assay of MMR-Cy5.5 in apoE-/- mice. (**a**) C57BL/6 nude mice (n = 5) were injected with 10 mg/kg of MMR-Cy5.5 via the tail vein, and whole body NIRF images were taken. (**b**) A total of 21 wild type C57BL/6 mice were injected with 10 mg/kg of MMR-Cy5.5 via the tail vein. Major organs such as the liver, lung, kidney, spleen, and heart were excised at scheduled time-points. Excised organs were imaged with IVIS 200. **(c)** Wild type C57BL/6 mice (n = 3 for each group) were injected with MMR-Cy5.5 or NT-Cy5.5 at a dose of 10 mg/kg via the tail vein. 3 non-injected mice were served as control. At 24 h post-injection, the harvested liver was imaged by *ex vivo* FRI and FM. Immunohistochemical staining for mannose receptors was followed. (d) Wild type C57BL/6 mice (n = 3) were injected with 10 mg/kg of MMR-Cy5.5 via the tail vein. 72 h after injection, liver, spleen, and kidney were harvested and analyzed. The illustrations were provided by Ji Woong Kim, with permission.


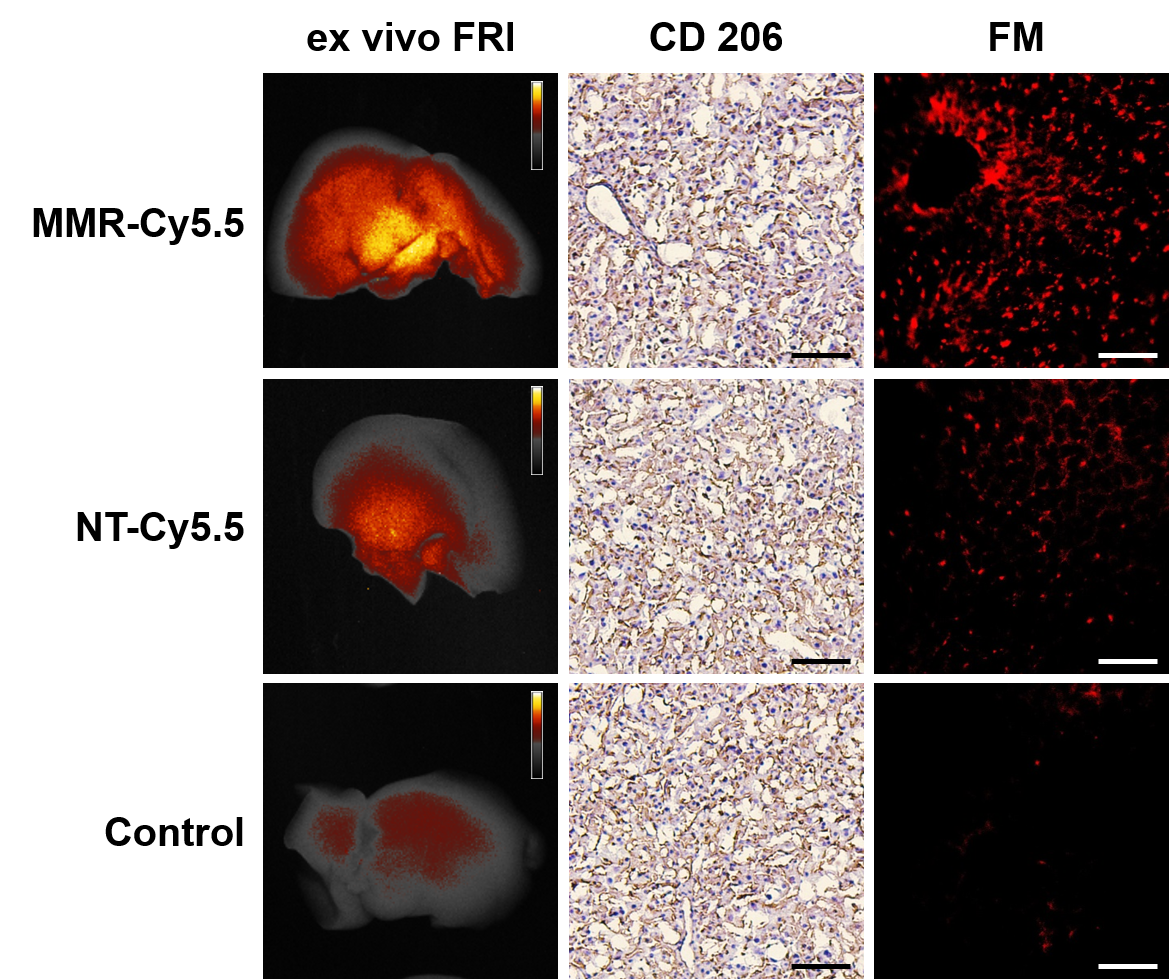


**Supplementary Fig 6. NIRF signal validation of excised liver.**

*Ex vivo* FRI images obtained 24 h after administration of MMR-Cy5.5 or NT-Cy5.5. While the expression of mannose receptors was similar between the groups, fluorescence signals were highest in the MMR-Cy5.5-injected group. FM imaging also demonstrated notably enhanced fluorescence signals in the MMR-Cy5.5-injected group compared to the other two. Scale bars, 100 μm. NIRF window is identical.


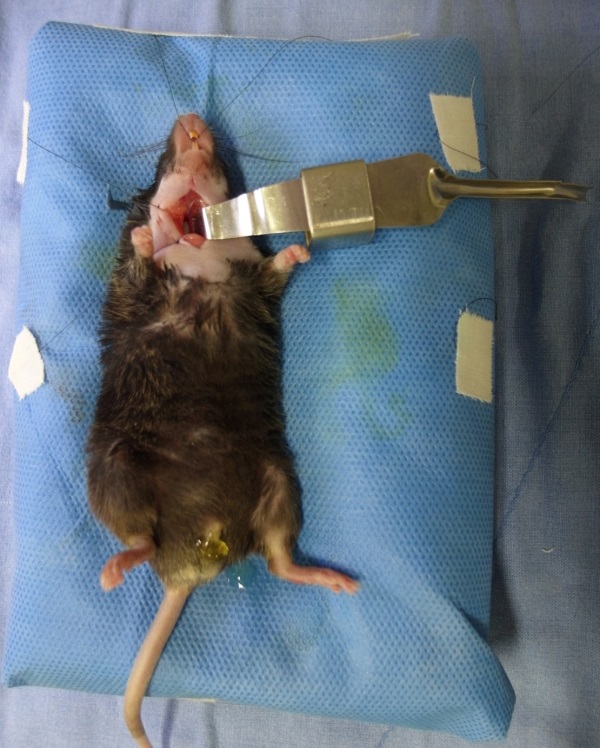


**Supplementary Fig 7. Mechanical Stabilization of Carotid Artery**

A metal plate derived from a palate knife was placed under the carotid artery to minimize the motion artifact coming from pulsation and respiratory movement. By the principle of the lever, the carotid artery was slightly elevated without significant disturbance of blood flow to counteract the disturbance induced from pulsation and respiratory movement, enabling high-resolution *in vivo* imaging.


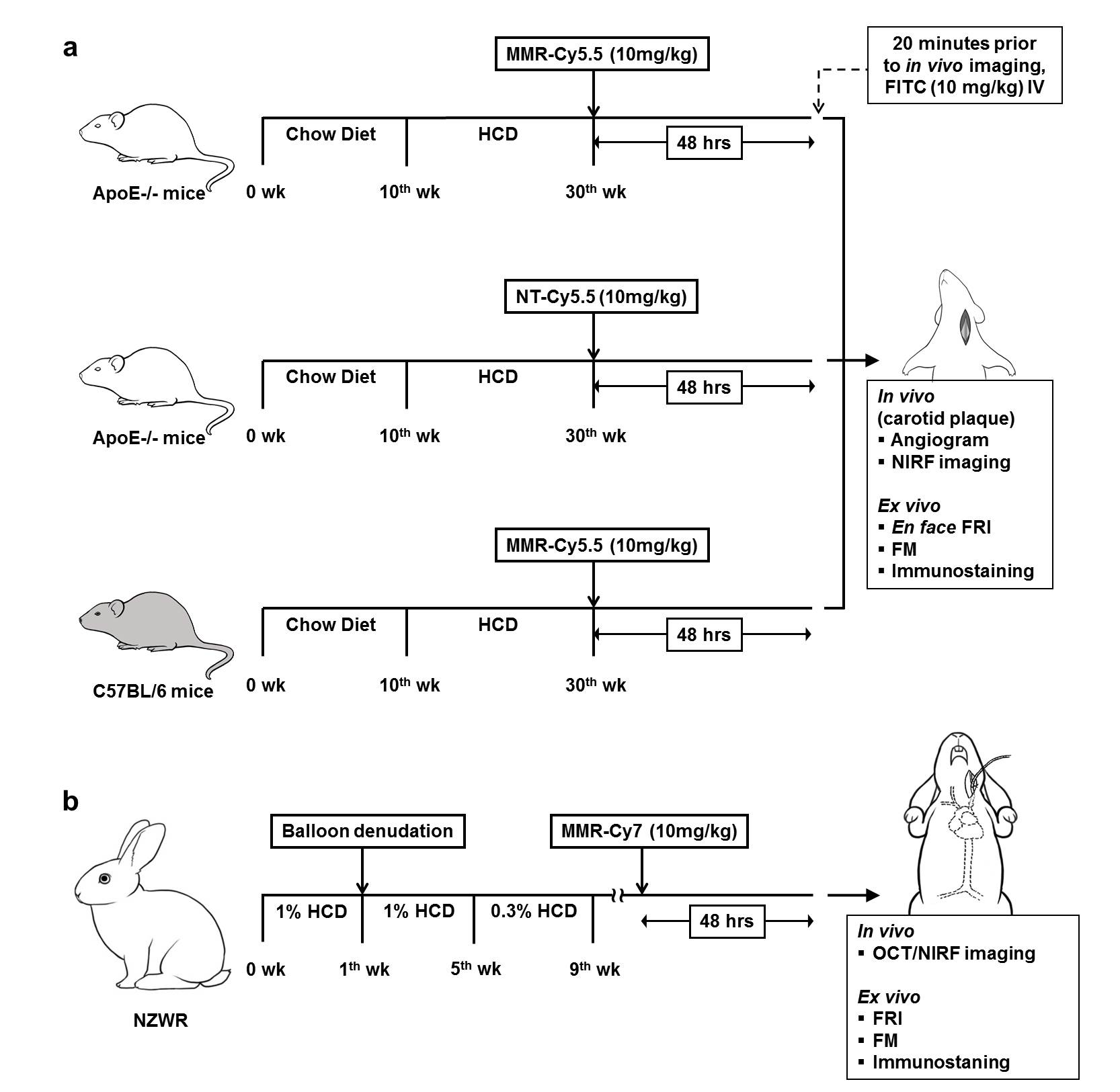


**Supplementary Fig 8. Experimental Protocol for the Animals**

*In vivo* study scheme for atherosclerotic mice and rabbits. **(a)** 10 week old apoE-/- mice (n = 12) were fed with high-cholesterol diet for 20 weeks, and then injected intravenously with 10 mg/kg of MMR-Cy 5.5, 48 h prior to imaging study. As control groups, age-matched apoE -/- mice and C57BL/6 mice were fed in the same way and injected with NT-Cy5.5 (n = 3) and MMR-Cy5.5 (n = 5), respectively. 20 min before the imaging, FITC-dextran (10 mg/kg, MW 2,000,000) was injected intravenously for angiogram. After *in vivo* imaging study of the left carotid artery of mice, carotid arteries were harvested for histologic analysis. (**b**) After 1 week of standard chow diet, New Zealand White Rabbits underwent balloon denudation followed by 8 weeks of high-cholesterol diet. 48 h prior to OCT-NIRF imaging, MMR-Cy7 was intravenously injected at a dose of 10 mg/kg. After *in vivo* imaging, the aorta and both iliac arteries were excised for *ex vivo* FRI and histologic validation. The illustrations were provided by Ji Woong Kim, with permission.


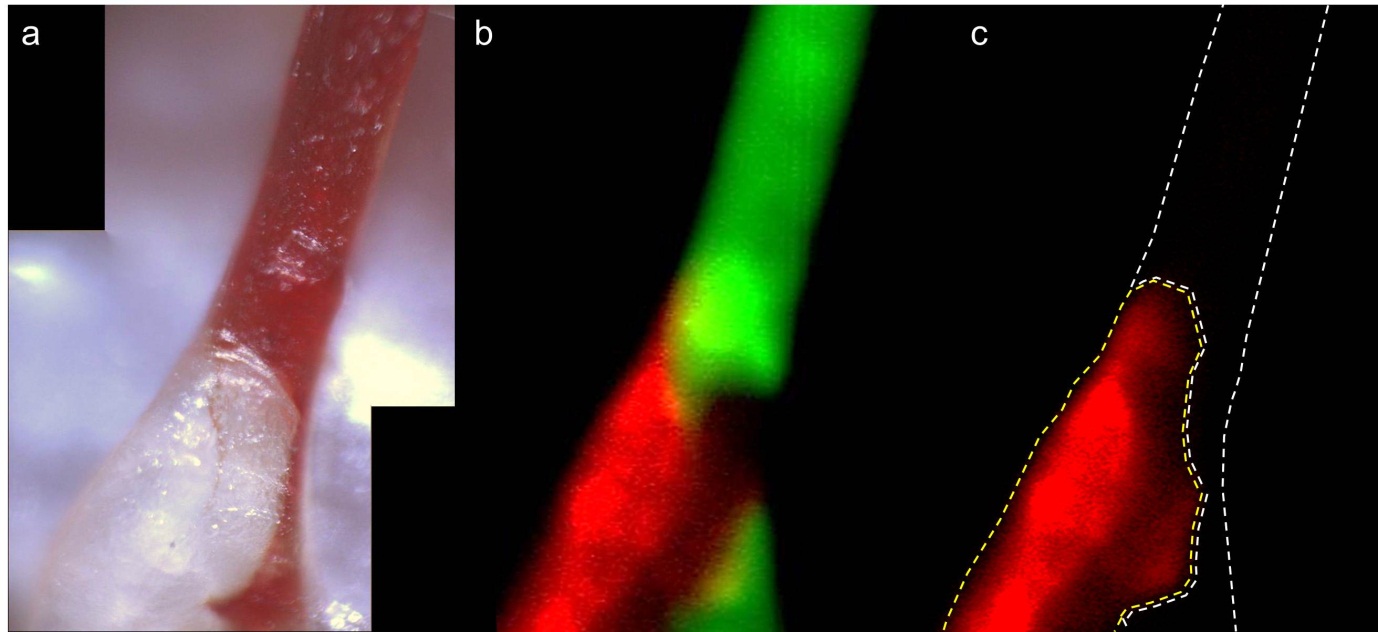


**Supplementary Fig 9. Measurement of Plaque TBR**

To compare signal intensity between groups of mice, plaque TBRs were calculated. (**a**) Plaque lesions and normal lesions were distinguished through the corresponding white light image. (**b**) After acquiring *in vivo* images and merging the two different channels of FITC (green) and Cy5.5 (red), (**c**) TBR was defined as: (ROI values from the atherosclerotic plaque; yellow dotted line) /(ROI values form the normal carotid artery; white dotted line).


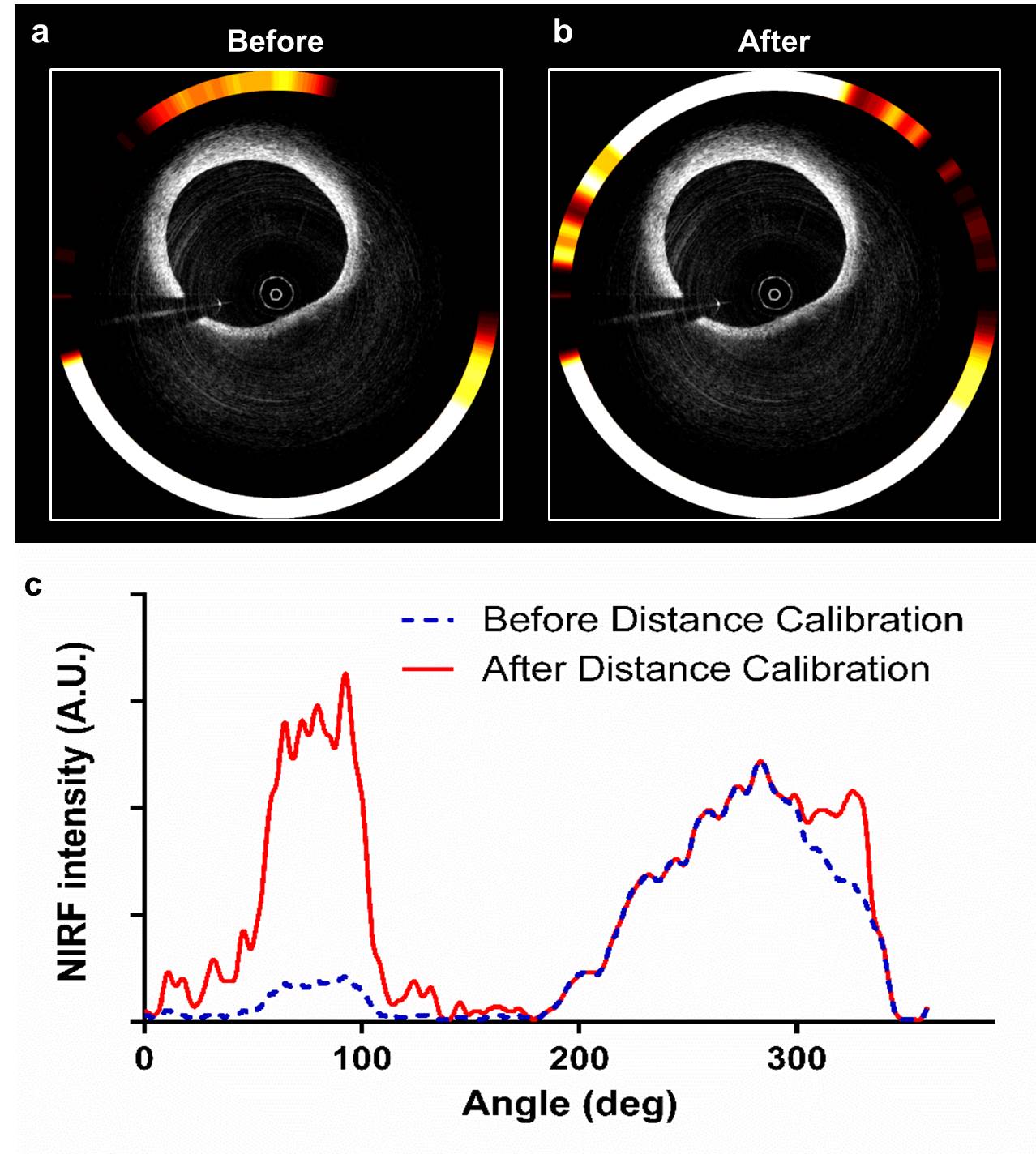


**Supplementary Fig 10.** **Distance Calibration of NIRF Signals**

NIRF signal intensity attenuates depending on the distance between the imaging catheter and the vessel wall. To compensate the attenuation and generate quantitative fluorescence signals, the signals were calibrated according to a previously established algorithm by our lab^1,2^. (**a**) An example of an OCT-NIRF image before distance calibration. Although a circumferential plaque exists according to the OCT, the NIRF signal intensity is weak in the upper half compared to the lower half of the plaque due to the relatively far distance between catheter and vessel wall. (**b**) After distance calibration, the calibrated NIRF signal intensity emitting from the upper half provides accurate acquisition of quantitative NIRF data. (**c**) Quantitative analysis of the NIRF signals before (blue dot line) and after (red line) distance calibration along the rotational direction. Note that the NIRF signal intensity augments from the angle of 0 to 150 degree which represents the upper half of the plaque, after distance calibration. A.U., Arbitrary Unit.


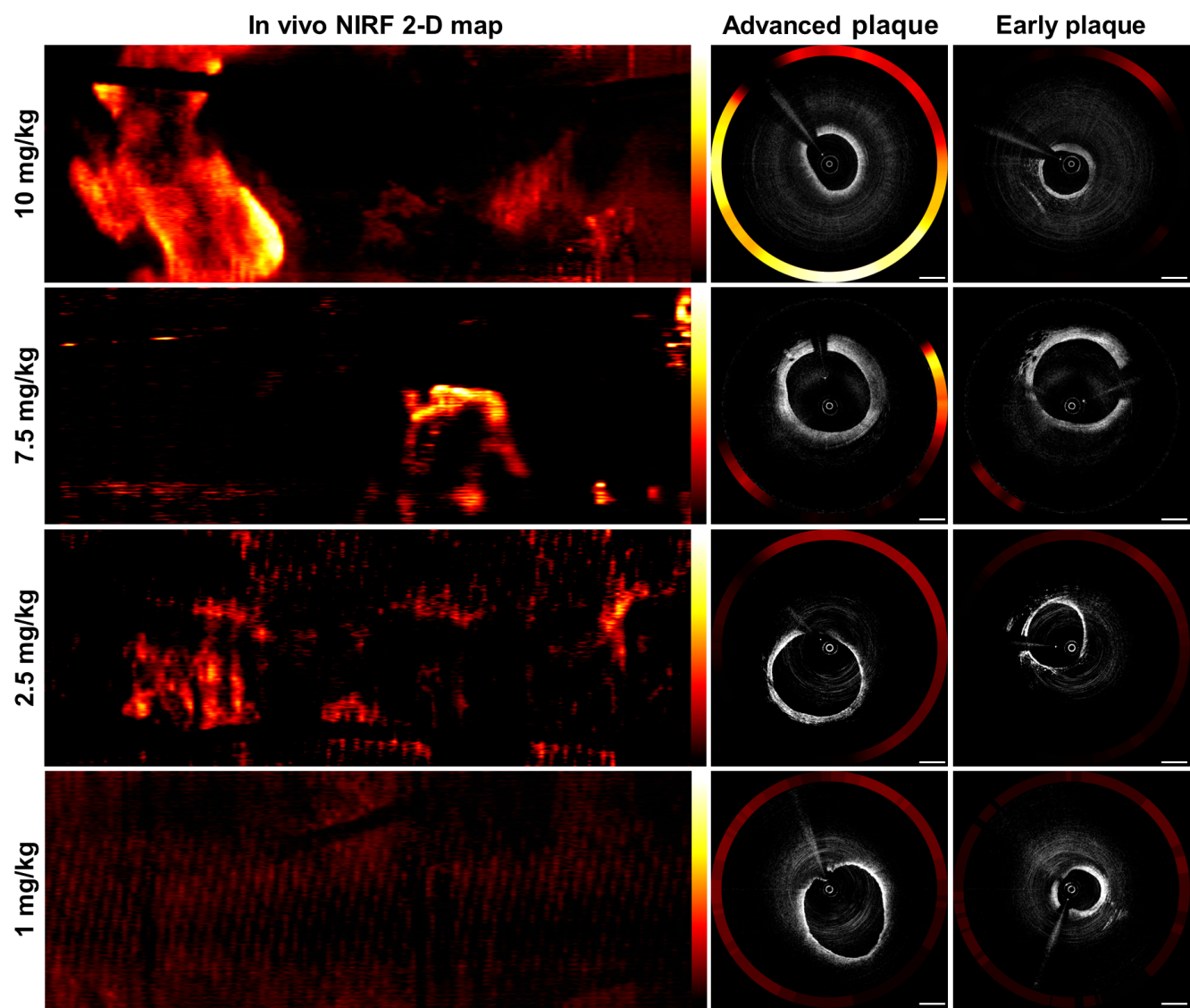


**Supplementary Fig 11.** **Assessment of Minimum MMR-Cy7 Dosage for High-risk Plaque Detection *In Vivo***

The minimum dose of MMR-Cy7 needed for *in vivo* detection of plaque macrophages using the OCT-NIRF imaging system was evaluated. 48 h after injection of MMR-Cy7 at the dosages of 1, 2.5, 7.5 and 10 mg/kg, intravascular OCT-NIRF imaging was performed. The representative *in vivo* images of an OCT-delineated advanced and early stage atherosclerotic plaque segments is presented. The OCT-NIRF catheter could detect fluorescence signals even at 1 mg/kg of MMR-Cy7 injection, however, the NIRF signals were very weak, not sufficient for clear identification of inflammatory plaque lesions. Thus, the dose to evidently identify inflamed high-risk plaques should be at least over 2.5 mg/kg. The same NIRF window was applied to all of the images above. Scale bars, 1 mm.


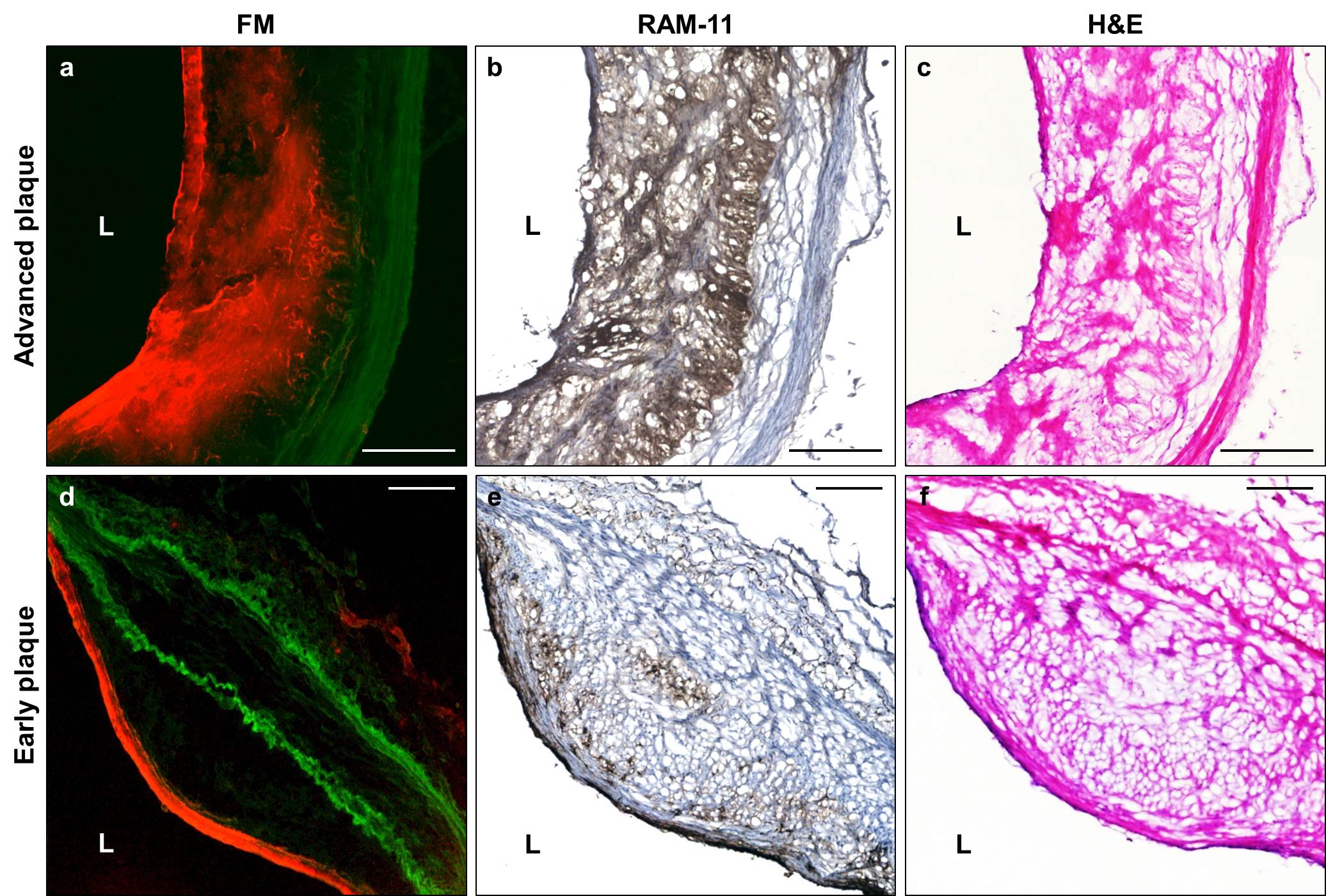


**Supplementary Fig 12. Penetration of MMR-targeting probes in Early to Advanced plaques**

(**a-c**) Histologic validation of NIRF signals emitting from advanced plaque lesions. The FM image demonstrates deep penetration into the neointimal layer through the disrupted fibrous cap presented by the H&E stained image, co-localizing well with macrophages stained with RAM-11. (**d-f**) In early plaque lesions, the NIRF signals are distributed mostly in the inner layer via binding to the macrophages in the intact fibrous cap. Scale bars, 200 μm.

**Supplementary Video 1. Three-dimensional reconstruction of carotid plaque**

*In vivo* confocal images obtained with the custom-built IVFM were three-dimensionally rendered. For every 5 μm-thick axial sections, 5 images were acquired and averaged to compensate the axial shift caused by the pulsation. Stacks of averaged images were collected along the vertical axis up to 150 μm, after which the acquired images were processed through imageJ software to produce a three-dimensional image of the carotid plaque. FITC and Cy5.5 signals are presented in green and red, respectively. The three-dimensional image represents sufficient penetration of the MMR-targeting NIRF probe into the atheroma and enables the approximate estimation of plaque volume in the carotid artery.

**Supplementary Video 2. Real-time OCT-NIRF Imaging**

*In vivo* real-time intravascular OCT-NIRF imaging of coronary-sized vessels in atheromatous rabbits injected with MMR-Cy7 starting at the distal descending aorta at a pullback speed of 20 mm/s. NIRF molecular imaging shows strong signals in atheromatous segments, whose morphology is simultaneously visualized by the OCT.

**Supplementary Video 3. Three-dimensional Flythrough Image**

Three-dimensional flythrough image rendered from an OCT-NIRF data set obtained from *in vivo* imaging of an atheromatous coronary-sized artery. The arterial wall on OCT imaging was color-coded in grey and the NIRF signals in red-yellow. The NIRF signals were overlaid on the luminal surface, providing complementary information regarding plaque inflammation and luminal stenosis.

**SUPPLEMENTARY REFERENCES**

1. Yoo, H.*, et al.* Intra-arterial catheter for simultaneous microstructural and molecular imaging in vivo. *Nat. Med.* **17**, 1680-1684 (2011).

2. Lee, S.*, et al.* Fully integrated high-speed intravascular optical coherence tomography/near-infrared fluorescence structural/molecular imaging in vivo using a clinically available near-infrared fluorescence-emitting indocyanine green to detect inflamed lipid-rich atheromata in coronary-sized vessels. *Circ. Cardiovasc. Interv.* **7**, 560-569 (2014).
